# Association of Fascin and matrix metalloproteinase-9 expression with poor prognostic parameters in breast carcinoma of Egyptian women

**DOI:** 10.1186/1746-1596-9-136

**Published:** 2014-07-04

**Authors:** Nermeen Salah Youssef, Sarah Adel Hakim

**Affiliations:** 1 Department of Pathology, Faculty of Medicine, Ain Shams University, Abbasseya square, Cairo, Egypt

**Keywords:** Breast carcinoma, MMP-9, Fascin, Immunohistochemistry

## Abstract

**Abstract:**

**Background**

The effects of fascin on cell invasiveness involve changes in cell motility and matrix metalloproteinase-9 (MMP-9) activity. Previous studies on the prognostic value of fascin and MMP-9 in breast carcinoma revealed conflicting results. To date, no immunohistochemical studies have been performed to assess the possible association between them in breast carcinoma. This study is designed to correlate their expression with prognostic parameters in breast carcinoma and assess the relationship between them.

**Methods**

Immunohistochemical expression of fascin and MMP-9 was evaluated semi quantitatively in 67 cases of breast carcinoma regarding the percentage of positive cells. Chi square test and Fisher’s exact test were used to examine the relationship between categorical variables. Kappa statistics was used to compute the measure of agreement between two investigational methods.

**Results**

Fascin and MMP-9 expressions were detected in 43.28% and 50.75% of breast carcinomas (respectively). Regarding the normal breast tissue, fascin expression was observed in myoepithelial cells and luminal cells of few ducts and acini. However, normal tissue showed negative MMP-9 expression. A significant relationship was observed between fascin and MMP-9 expression and lymph node metastases (p = 0.001 and 0.002 respectively), advanced tumor stage (p = 0.004 and 0.005 respectively), estrogen receptor negative (p = 0.002 and 0.005 respectively), progesterone receptor negative (p = 0.001 and 0.003 respectively) hormonal status and molecular subtypes (p = 0.0007 and 0.014 respectively). A significant strong agreement was detected between fascin and MMP-9 expression (p = 0.0001). More intense immunostaining of fascin and MMP-9 was observed at the invasive fronts compared with other areas of the tumor. Moreover, a significant moderate agreement between fascin and MMP-9 was found regarding the site of predominant intensity.

**Conclusion**

Fascin and MMP-9 proteins are associated with parameters of poor prognosis in breast cancer. The significant strong agreement between the two markers supports the role of fascin in cell invasiveness by activating matrix proteases besides increasing cell motility. Both proteins may represent potential therapeutic targets for patients with breast cancer especially those with hormone receptor–negative status.

**Virtual slides:**

The virtual slide(s) for this article can be found here: http://www.diagnosticpathology.diagnomx.eu/vs/1421167695121127.

## Background

Breast cancer is one of the leading causes of cancer mortality in women worldwide. In spite of significant advances in cancer treatment, mortality results from local invasion and/or distant metastasis and not from the tumor in the primary site [1]. Tumor invasion and metastasis are the results of several sequential steps and in part are caused by the highly motile properties of tumor cells to overcome cell-cell and cell matrix adhesion and to invade surrounding tissue. Invasive tumor cells often show specific morphologic features, such as the appearance of membrane protrusions as well as loss of cell-cell adhesion and loss of junctional communications. These features are thought to result from rearrangements of the cytoskeletal microfilaments by the action of actin cross-linking proteins [2].

Fascin, also known as fascin-1, is a 55-kDa globular actin bundling protein [3] originally found in the extracts of unfertilized sea urchin eggs and localized to the microfilament bundles within microvilli cores and within filo podia on the surface of fertilized sea urchin eggs [4]. In mammalian cells, fascin is present in membrane ruffles, micro spikes, and other motility-associated cell fibers [5]. It is a key regulator of the actin cytoskeleton and is the leading regulator of filament bundling for the formation of filopodia, which are actin-based protrusive sensory organelles that contribute to the initiation of cell movement and cell migration [4].

Fascin, is normally expressed in neuronal and mesenchymal cells and is low or absent in epithelia [4,6]. However, striking up-regulation of fascin has been reported in several human malignant epithelial tumors including colon, urinary bladder, pancreatic and lung carcinomas [7-10]. Most of the immunohistochemical studies have shown that fascin expression is correlated with the clinical aggressiveness of tumors and with poor patient survival [11]. Its down regulation reduces cell motility and invasiveness in esophageal squamous cell carcinoma [12]. However, it is not yet clear if fascin has an independent value as a biomarker or not, because individual studies are not always consistent [13]. To date, only few immunohistochemical studies have been conducted on fascin expression in breast carcinomas. Such studies have reached discrepant conclusions on their association with clinicopathological prognostic parameters and metastasis [14-17].

A recent study using HCC (hepatocellular carcinoma) cell lines showed that the migratory effect of fascin-1 on HCC cells led to efficient invasion when assisted with secretory factors from intrinsically highly invasive cells such as MMP-2 (matrix metalloproteinase-2) and MMP-9, which fascin-1 alone could not up regulate [18]. It has been previously reported that tumor necrosis factor α-induced MMP-9 production in cholangiocarcinoma (CC) cells is concurrent with enhanced fascin-1 expression. As silencing of fascin-1expression abrogated MMP-9 induction, fascin-1 was proposed be involved in the signaling pathway for tumor necrosis factor α -related MMP-9 production [19]. The effects of fascin on cell invasiveness involve both changes in cell motility as well as the activity of matrix proteases [20].

Matrix metalloproteinases (MMPs), potent proteolytic enzymes are known to play key roles in degradation of basement membranes and extracellular matrix [21]. Within the MMP families, MMP-9 (92 kDa type IV collaganase, gelatinase B) is able to degrade type IV collagen that is abundant in basement membranes separating the epithelial cells from the underlying stroma. Increased expression and activity of MMP - 9 in tumors lead to the degradation of basement membranes, an essential step in invasion and metastasis of malignant tumors [22]. Previous studies on the prognostic value of MMP-9 expression in human cancers including breast carcinoma revealed conflicting results [23-26].

To the best of our knowledge, no immunohistochemical studies have been performed to assess the possible association between fascin and MMP-9 in breast carcinoma. This study is designed to correlate fascin and MMP-9 expression with clinicopathological prognostic parameters in breast carcinoma and assess the relationship between these two proteins.

## Methods

### Tissue and patient data

The current study was conducted on 67 cases of invasive ductal breast carcinoma which is the most common subtype of breast carcinomas. Cases were obtained from the Archives of the Pathology Lab. of Ain-Shams University Specialized Hospital. Such cases were diagnosed during the period from January 2010 to January 2012. They were obtained by modified radical mastectomy. The surgical and histopathology reports were reviewed to determine age of patients, tumor size (greatest dimension), estrogen-receptor (ER), progesterone-receptor (PR) and HER2 status, as well as lymph nodal involvement. For each patient, clinical stage at presentation was classified according to the 2003 American Joint Committee on Cancer Staging System [27]. Haematoxylin and Eosin stained slides were examined to re-evaluate and verify the histopathologic diagnosis and grade (according to the modified Bloom and Richardson method [28]). Only cases with information for all the covariates were selected in the analysis.

### Ethics statement

All patients who participated in this study signed a written, informed consent before surgery. The study was approved by the Research Ethical Committee at Faculty of Medicine, Ain Shams University.

### Immunohistochemical staining

Four micrometer sections of formalin –fixed and paraffin- embedded samples of 67 breast carcinoma cases were prepared. They included the tumor and the adjacent normal breast tissue. Immunohistochemical staining was performed using primary antibodies; mouse monoclonal anti-fascin (Clone: FCN01; 1:200 dilution; Thermo Fisher Scientific Inc., Fremont, CA) and mouse monoclonal anti MMP-9 (Clone: GE-213;1:200 dilution; Thermo Fisher Scientific Inc., Fremont, CA). Avidin-Biotin immunoperoxidase complex technique was used according to Hsu et al. [29] by applying the super sensitive detection kit (Biogenex, CA, USA). The prepared tissue sections were fixed on poly-L- lysine coated slides overnight at 37°C. They were deparaffinized and rehydrated through graded alcohol series. Then the sections were heated in a microwave oven in 10 mM citrate buffer (pH 6.0) for 20 min. After the blocking of endogenous peroxidase and incubation in Protein Block Serum-Free Solution (Dako Cytomation) for 20 min, the sections were incubated overnight at 4°C with primary antibodies. Biotinylated anti- mouse immunoglobulin and streptavidin conjugated to horseradish peroxidase were then added. Finally, 3,3′ – diaminobenzidine as the substrate or chromogen was used to form an insoluble brown product. Finally, the sections were counterstained with hematoxylin and mounted. Sections of Hodgkin’s disease and placenta were used as positive control for fascin and MMP-9 respectively. Negative control sections were incubated with normal mouse serum instead of the primary antibody.

### Interpretation of immunohistochemical staining

Immunohistochemical analysis of fascin and MMP-9 was blindly performed by the two pathologists (the authors) without any prior knowledge of the clinicopathological data. Any discrepancies were resolved by consensus using a multi-headed microscope. Cytoplasmic expression of fascin and MMP-9 was evaluated semi-quantitatively according to the percentage of positive cells in at least five areas at a magnification of 400×, and assigned to one of the four following categories: 0, negative; 1, focally positive (1–10% positive cells in the lesion); 2, moderately positive (11–50%); and 3, markedly positive (more than 50%). Cases with moderate or marked expression patterns (scores 2 or 3) were considered positive cases in this study [19]. The intensity of expression of both proteins was assessed on the basis of the predominant area of intensity (invasive fronts of the tumor, invasive fronts and other areas or only other areas).

Using immunohistochemistry for ER, PR, and HER2 as a surrogate for expression profiling, the studied tumors were classified according to Cheng et al. [30] as follows:

(a) Triple negative subtype [ER-, PR-, and HER2 -]

(b) HER2 subtype [HER2+/ER-PR-].

(c) Luminal A subtype [ER + and/or PR + plus HER2- with histologic grade 1 or 2].

(d) Luminal B subtype [ER + and/or PR + plus HER2+ or ER + and/or PR + plus HER2- with histologic grade 3].

### Statistical analysis

Continuous variables are expressed as mean and Standard Deviation. Categorical variables are expressed as frequencies and percent. Chi square and Fisher’s exact test were used to examine the relationship between categorical variables. Kappa statistics was used to compute the measure of agreement between two investigational methods; Kappa’s values < 0 indicated no agreement while 0–0.20 indicated slight agreement, 0.21–0.40: fair, 0.41–0.60: moderate, 0.61–0.80: strong and 0.81–1 revealed almost perfect agreement. P < 0.05 was considered the cut-off value of significance. All statistical procedures were carried out using SPSS version 15 for Windows (SPSS Inc, Chicago, IL, USA).

## Results

Clinical and pathological data for the studied breast carcinoma cases are represented in Table 1. All the patients are females, and their mean age is 55.3 years (Standard deviation, ± 11.3 ; range, 35–76 years).

**Table 1:** Relationship between fascin and MMP-9 expressions and clinicopathological parameters of the studied breast carcinomas (n = 67)

		**Fascin expression**			**MMP-9 expression**		
**Variable**	**n (%)**	**Negative n = 38**	**Positive n = 29**	**P value**	**Negative n = 33**	**Positive n = 33**	**P value**
***Age (years)***							
≤ 50	22 (33)	10 (45.5%)	12 (54.5%)	0.193*	10 (45.5%)	12 (54.5%)	0.664*
>50	45 (67)	28 (62.2%)	17 (37.8%)	(NS)	23 (51.1%)	22 (48.9%)	(NS)
***Tumor size (cm)***							
≤5	41 (61.19)	27 (65.9%)	14 (34.1%)	0.058*	24 (58.5%)	17 (41.5%)	0.056*
>5	26 (38.81)	11 (42.3%)	15 (57.7%)	(NS)	9 (34.6%)	17 (65.4%)	(NS)
***Grades***							
I	7 (10.4)	3 (42.9%)	4 (57.1%)	0.111**	2 (28.6%)	5 (71.4%)	0.088**
II	44 (65.7)	29 (65.9%)	15 (34.1%)	(NS)	26 (59.1%)	18 (40.9%)	(NS)
III	16 (23.9)	6 (37.5%)	10 (62.5%)		5 (31.2%)	11 (68.8%)	
***Lymph nodal status***							
Node –	24 (35.8)	20 (83.3%)	4 (16.7%)	0.001*	18 (75.0%)	6 (25.0%)	0.002*
Node +	43 (64.2)	18 (41.9%)	25 (58.1%)	(S)	15 (34.9%)	28 (65.1%)	(S)
***Stage***							
I	18 (26.87)	15 (83.3%)	3 (16.7%)	0.004*	14 (77.8%)	4 (22.2%)	0.005*
II	29 (43.28)	17 (58.6%)	12 (41.4%)	(S)	14 (48.3%)	15 (51.7%)	(S)
III	20 (29.85)	6 (30.0%)	14 (70.0%)		5 (25.0%)	15 (75.0%)	
***Estrogen receptor***							
Negative	21 (31.3)	6 (28.6%)	15 (71.4%)	0.002*	5 (23.8%)	16 (76.2%)	0.005*
Positive	46 (68.7)	32 (69.6%)	14 (30.4%)	(S)	28 (60.9%)	18 (39.1%)	(S)
***Progesterone receptor***							
Negative	24 (35.8)	7 (29.2%)	17 (70.8%)	0.001*	6 (25.0%)	18 (75.0%)	0.003*
Positive	43 (64.2)	31 (72.1%)	12 (27.9%)	(S)	27 (62.8%)	16 (37.2%)	(S)
***HER2***							
Negative	55 (82.1)	33 (60.0%)	22 (40.0%)	0.246*	30 (54.5%)	25 (45.5%)	0.064*
Positive	12 (17.9)	5 (41.7%)	7 (58.3%)	(NS)	3 (25.0%)	9 (75.0%)	(NS)

*Chi-square test.

**Fisher exact test.

S: significant.

NS: non-significant.

n = number of cases.

### Expression of fascin and its relationship with clinicopathological parameters

**Figure 1:**
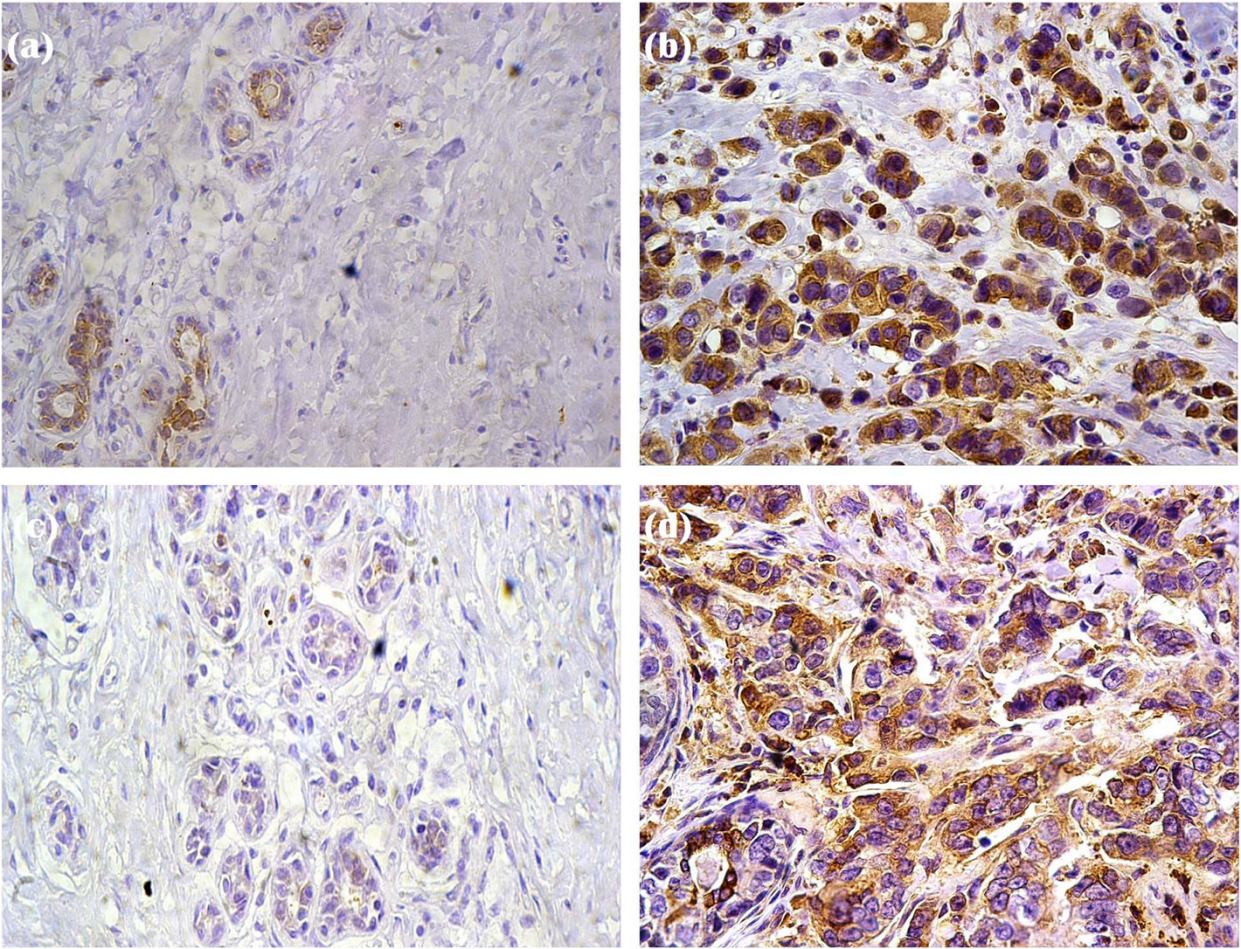
**Fascin and MMP-9 expressions in normal breast tissue and invasive ductal carcinomas. a**: Moderate fascin expression in the myoepithelial cells and luminal cells of few normal acini (IHC × 400). **b**: Positive cytoplasmic fascin expression in malignant cells (IHC × 400). **c**: Negative MMP-9 expression in normal breast tissue (IHC × 400). **d**: Positive cytoplasmic MMP-9 expression of malignant cells with weak expression of stromal cells (IHC × 400).

**Figure 2:**
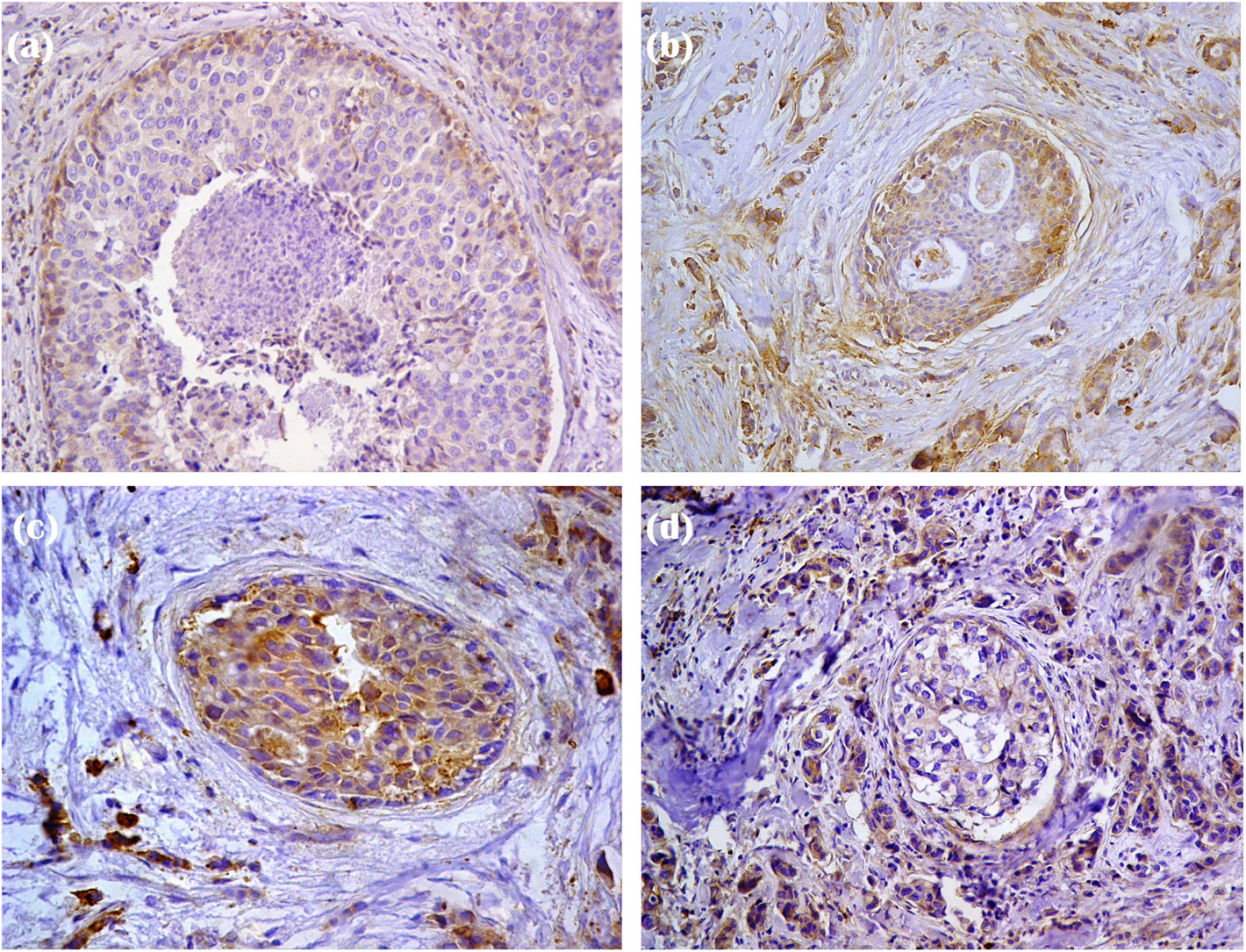
**Fascin and MMP-9 expression in invasive ductal carcinomas with adjacent in situ component. (a)**: Positive cytoplasmic fascin expression in the myoepithelial cells of the in situ component (IHC × 200). **(b)**: Positive cytoplasmic fascin expression in the in situ carcinoma lesions as well as the adjacent invasive carcinoma (IHC × 200). **(c)**: Positive cytoplasmic MMP-9 expression in the in situ carcinoma lesions (IHC × 200). **(d)**: Positive cytoplasmic MMP-9 expression in invasive carcinoma and negative expression in the in situ carcinoma lesions (IHC × 200).

**Figure 3:**
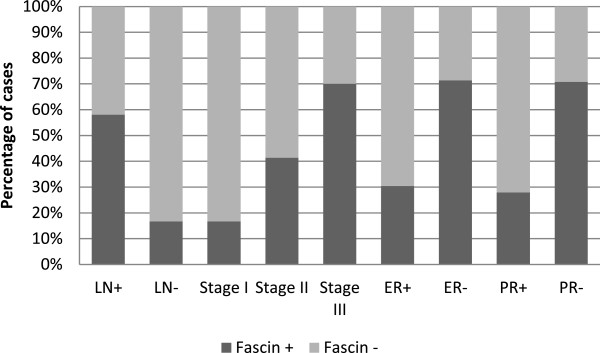
Relationship between fascin expression and poor prognostic parameters in breast carcinoma.

In normal breast tissue, fascin expression was observed in myoepithelial cells and luminal cells of few ducts and acini (Figure 1a). Eight (12%) out of 67cases of invasive ductal carcinomas showed adjacent ductal carcinoma in situ (DCIS). Fascin expression was present in myoepithelial cells of the in situ component (Figure 2a). Moreover, out of the eight cases, only two cases (25%) revealed fascin expression in ductal epithelial cells of in situ carcinoma lesions (Figure 2b). However, 29 (43.28%) out of 67 invasive ductal breast carcinomas exhibited cytoplasmic fascin expression (Figures 1b and 2b). Fascin was expressed in endothelial cells of the stroma surrounding neoplastic epithelial cells. Moreover, it was also expressed by histiocytes and lymphoid cells. There was a statistical significant relationship between fascin expression and lymph node metastases (p = 0.001) and advanced tumor stage (p = 0.004). Moreover, there was an inverse correlation between fascin expression and both estrogen receptor (p = 0.002) and progesterone receptor (p = 0.001) hormonal status. However, no statistical correlation was found between fascin expression and patients’ age (p = 0.193), tumor size (p = 0.058), histological grade (p = 0.111) or HER2 status (p = 0.246) (Figure 3). The relationship between fascin expression and clinicopathological parameters is shown in Table 1.

### Expression of MMP-9 and its relationship with clinicopathological parameters

**Figure 4:**
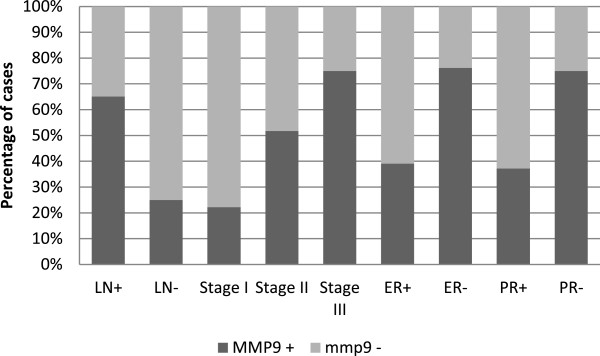
Relationship between MMP-9 expression and poor prognostic parameters in breast carcinoma.

The normal breast tissue adjacent to the tumor was negative for MMP-9 expression (Figure 1c). Three (37.5%) out of the eight cases of invasive ductal carcinoma with adjacent in situ ductal component revealed positive MMP-9 expression in the in situ carcinoma lesions (Figure 2c) while the rest of the cases showed negative expression (Figure 2d). The expression pattern of MMP-9 was cytoplasmic in both cancer cells and stromal cells (Figure 1d). However, MMP-9 staining in stromal cells was weak; therefore its staining in stromal cells was not assessed. Thirty four (50.75%) out of 67 breast carcinoma samples were immunoreactive for MMP-9. There was a significant correlation between MMP-9 expression and lymph node metastases (p = 0.002), advanced tumor stage (p = 0.005) as well as both estrogen receptor negative (p = 0.005) and progesterone receptor negative (p = 0.003) hormonal status (Figure 4). However, no statistical association was detected between MMP-9 expression and patients’ age (p = 0.664), tumor size (p = 0.056), histological grade (p = 0.088) or HER2 status (p = 0.064). These data are also summarized in Table 1.

### Relationship between fascin and MMP-9expression and breast cancer molecular subtypes

Triple negative subtype and HER2 subtype showed a significant higher rate of fascin and MMP-9 expression than luminal subtypes (A&B) (p = 0.0007 and 0.014 respectively). Data for fascin and MMP-9 expression in different breast cancer molecular subtypes are summarized in Table 2.

**Table 2:** Relationship between fascin and MMP-9 expression and breast cancer molecular subtypes (n = 67)

**Molecular subtype**		**Fascin expression**			**MMP-9 expression**		
		**Positive**	**Negative**	**P-value**	**Positive**	**Negative**	**P-value**
		**n (%)**	**n (%)**		**n (%)**	**n (%)**	
Triple negative	12 (17.9)	11 (91.7)	1 (8.3)	0.0007* (S)	10 (83.3)	2 (16.7)	0.0143* (S)
HER2 subtype	9 (13.4)	4 (44.4)	5 (55.6)		6 (66.7)	3 (33.3)	
Luminal subtype	46	14 (30.4)	32 (69.6)		18 (39.1)	28 (60.9)	
*-Luminal A*	*33 (49.3)*						
*-Luminal B*	*13 (19.4)*						

*Chi-square test.

S: significant.

n = number of cases.

### Agreement between fascin and MMP-9 expressions in breast carcinoma

**Figure 5:**
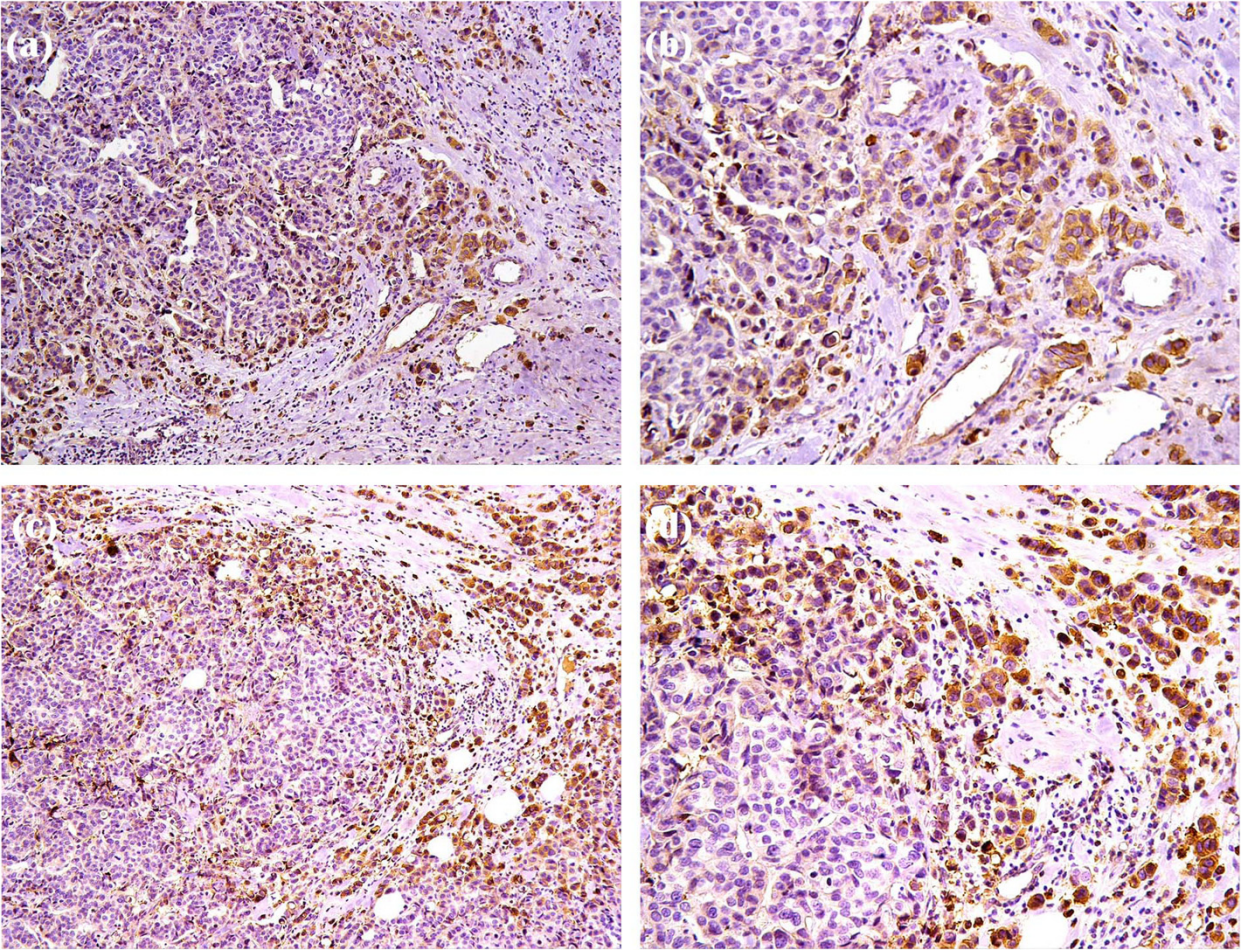
**Invasive ductal breast carcinomas. a**: Enhanced fascin expression at the leading edge of the tumor (IHC × 100). **b**: The same field with higher magnification power showing positive cytoplasmic fascin expression of malignant cells and endothelial cells (IHC × 200). **c**: Enhanced MMP-9 expression at the tumor–host border (IHC × 100). **d**: The same field with higher magnification power showing positive cytoplasmic MMP-9 expression of malignant cells (IHC × 200).

Comparing fascin and MMP-9 expressions in each case, out of 29 cases with positive fascin expression, 27 cases (93.1%) showed also positive MMP-9 immunoreactivity. There was a significant strong agreement between fascin and MMP-9 expressions (kappa = 0.723, p = 0.0001) (Table 3). Next, the intensities of immunohistochemical staining of fascin and MMP-9 were compared in the invasive front versus other areas in each tumor. As shown in Table 4, 75.9% of breast carcinomas with positive fascin expression showed more intense immunostaining at the invasive fronts compared with other areas (Figure 5a and b). Similarly, 67.7% of breast carcinoma samples showing positive MMP-9 expression were found to have intense expression at the invasive fronts (Figure 5c and d). Moreover, there was a significant moderate agreement between fascin and MMP-9 regarding the site of predominant intensity (kappa = 0.434, p = 0.012) (Table 5).

**Table 3:** Agreement between fascin and MMP-9 expressions in breast carcinoma cases (n = 67)

	**Fascin expression**			
	**Negative (n = 38)**	**Positive (n = 29)**	***Kappa***	***p- value***
MMP-9 expression				
Negative (n = 33)	31 (81.6%)	2 (6.9%)	0.723	0.0001
Positive (n = 34)	7 (18.4%)	27 (93.1%)		

n = number of cases.

*P-value* < 0.0001: very highly significant.

**Table 4:** Intensities of fascin and MMP-9 expressions in breast carcinoma cases

	**Intensity of expression on the basis of the predominant area**		
	**Invasive front > other areas**	**Invasive front = other areas**	**Invasive front < other areas**
Fascin expression (29)*	22 (75.9%)	6 (20.7%)	1 (3.4%)
MMP-9 expression (34)**	23 (67.7%)	10 (29.4%)	1 (2.9%)

*Breast carcinoma cases with positive fascin expression.

**Breast carcinoma cases with positive MMP-9 expression.

**Table 5:** Agreement between intensities of fascin and MMP-9 expressions in 27 cases positive for both proteins

		**Fascin expression**						***Kappa***	***P***
		**Invasive front < other areas**		**Invasive front = other areas**		**Invasive front > other areas**			
		**N**	**%**	**N**	**%**	**N**	**%**		
MMP-9	Invasive front < other areas	0	0.0%	1	3.7%	0	0.0%	0.434	0.012 (S)
	Invasive front = other areas	1	3.7%	4	14.8%	4	14.8%		
	Invasive front > other areas	0	0.0%	1	3.7%	16	59.3%		

## Discussion

Metastasis remains the main cause of cancer mortalities [1], stressing the need to understand the cellular and molecular mechanisms that regulate this process. It is a complex process where cytoskeletal proteins were reported to regulate multiple cellular processes including morphological changes and motility, which are critical steps for metastasis [31].

Fascin is an actin-bundling motility-associated protein that plays an important role in the assembly of cell-motility structures [32]. Therefore, it is possible that fascin expression in cancer cells may lead to a more clinically aggressive course through augmented cell motility and enhanced metastatic potential, a finding supported by in vitro observations [33]. Most of studies have shown that fascin expression is correlated with the clinical aggressiveness of tumors and with poor patient survival. In studies of breast cancer, some researchers have shown that fascin expression increases in more advanced tumors [15], but the results of other studies have not agreed with those findings [14].

In the current study, 43.28% of invasive ductal breast carcinomas showed positive fascin expression. However, in the adjacent normal breast tissue, fascin expression was observed in myoepithelial cells and luminal cells of few ducts and acini. These results are consistent with previous studies showing striking up-regulation of fascin in a variety of malignancies including breast carcinoma [7-10,17], while normal epithelia exhibited very low levels of expression [14,16]. In agreement with Onodera et al. [19], we found positive fascin expression in DCIS of 25% of invasive ductal carcinomas with in situ component. Only few studies, investigating fascin expression in breast cancer tissues, have been conducted showing different rates of expression. Grothey et al. [14] found that 70% of estrogen receptor negative and 50% of progesterone receptor negative breast cancer were positive for fascin expression. Yoder et al. [15] observed positive fascin immunoreactivity in 16% of the invasive breast carcinomas. Rodriguez-Pinilla et al. [16] detected fascin expression in 25.1% of sporadic invasive breast carcinomas and in 83.3% and 16.7% BRCA1- and BRCA2-associated carcinomas, respectively. Al Alwan et al. [17] demonstrated fascin in the tumor cells of 40.84% of invasive ductal breast carcinoma patients.

Heterogeneity in the results was apparent within breast carcinoma studies. This could be due to difference in the commercial company supplying the primary antibody, and the method of immunohistochemical staining. Furthermore, it should be noted that each study included different histological types of breast carcinomas. Grothey et al. [14] investigated hormone receptor -negative breast cancer. Yoder et al. [15] studied primary node-positive and node-negative invasive breast carcinomas, which included infiltrating ductal carcinomas, infiltrating lobular carcinomas, mucinous carcinomas and medullary carcinomas. Rodriguez-Pinilla et al. [16] investigated node-negative sporadic and hereditary invasive breast carcinomas. Al-Alwan et al. [17] as well as our current research studied invasive ductal carcinoma only.

In our work, we found a significant relationship between fascin expression and negative prognostic factors as lymph node metastases and advanced tumor stage. Concomitant with these results, Al Alwan et al. [17] found a significant correlation between fascin expression and lymph nodal metastases and Yoder et al. [15] reported a significant association between fascin expression and advanced tumor stage. However, Yoder et al. [15] as well as Grothey et al. [14] did not find any significant relationship between fascin expression and lymph nodal status. Moreover, the later [14] did not find any correlation with advanced tumor stage.

The present research confirms a statistically significant inverse relationship between fascin expression and estrogen receptor and progesterone receptor status. These observations are in line with previous studies [14-17]. Hormone receptor–negative breast cancers traditionally have a worse prognosis and fewer available treatment options (ineffectiveness of hormonal therapy) compared with hormone receptor–positive tumors [34-36]. It is interesting that hormone receptor–negative breast cancers also display increased cell motility in vitro [37,38]. In a study examining the ability of breast cancer cell lines to penetrate into a collagen-fibroblast matrix, cells expressing mRNA for estrogen receptor showed a noninvasive phenotype, whereas cells lacking estrogen receptor mRNA were shown to be highly invasive [38]. Yoder et al. [15] suggested a connection between the expression of fascin and the absence of hormone receptors, increased cell motility, and decreased survival in human breast cancers. It is conceivable that fascin may serve as a downstream cytoskeletal effector contributing to the more aggressive/malignant phenotype of hormone receptor -negative breast cancer.

Interestingly, no association was identified in the present study between fascin expression and patients’ age or tumor size. These observations are in accordance with Yoder et al. [15] and Rodriguez et al. [16]. However, in a recent study, fascin correlated significantly with tumor size [17].

There was no significant relationship between fascin immunoreactivity and tumor grade. A similar absence of correlation has also been noted by Grothey et al. [14] and Al- Alwan et al. [17] although Yoder et al. [15] and Rodriguez et al. [16] reported a significant positive association between fascin expression and tumor grade.

In vitro, studies revealed that fascin exhibits highly increased levels in breast cancer cell lines over-expressing the receptor tyrosine kinase and prognostic indicator c-erbB-2/HER-2, and that such cells exhibit dramatically increased cell dynamics and in vitro motility [39]. The data presented in our research failed to reveal any association between fascin and HER2 status in tissue samples concomitant with Yoder et al. [15], Rodriguez et al. [16] and Al- Alwan et al. [17]. This could be due to limited population size or an institutional bias. Alternatively, the forced overexpression of this receptor in cell cultures by transfection may represent an artificial system, which may not well reflect the biological complexity of HER2 gene amplification and protein overexpression occurring in vivo.

By using siRNA technology, Xie et al. [20] have successfully silenced fascin gene in EC109 cells, an esophageal squamous cell carcinoma cell line. They found that decreased level of fascin correlated with decreased formation of surface protrusions that play essential roles in cell motility. Besides the decreased formation of protrusions, Xie et al. [20] suggested another possible mechanism for fascin effect on cell invasiveness, as shown in the gelatin zymography, which was the decreased activity of extracellular matrix proteases as MMP-2 and MMP-9. Such proteases digest collagen type IV and other components of the basement membrane and play a key role in local invasiveness and the formation of distant metastases by malignant tumors [22]. Xie et al. [20] postulated that the effects of fascin on cell invasiveness involve both changes in cell motility as well as the activity of matrix proteases. Furthermore, Onodera et al. [19] showed that fascin was responsible for the overproduction of MMP-9 in cholangiocarcinoma (CC), raising a possibility that fascin relates not only to increased cell motility but also to stromal degradation during the invasion of CC. In addition, the migratory effect of fascin-1 on hepatocellular carcinoma cells led to efficient invasion when assisted with secretory factors from intrinsically highly invasive cells such as MMP-9, which fascin-1 alone could not up regulate. Concomitant with these observations, our study revealed a significant strong agreement between fascin and MMP-9 expressions in breast carcinoma cases. To the best of our knowledge, this is the first study to investigate the relationship between immunohistochemical expression of fascin and MMP-9 in breast cancer.

In the current research more intense expression of fascin and MMP-9 was observed at the invasive fronts compared with other areas of tumor. Moreover, a significant moderate agreement between fascin and MMP-9 was found regarding the site of predominant intensity. These observations are in agreement with Onodera et al. [19]. In addition, Grothey et al. [14] previously observed that fascin staining is often enhanced at the leading edges of infiltrating tumors, which indicates its role as a pathogenic factor for tumor cell invasion. Onodera et al. [19] demonstrated that overexpression of MMP-9 induced by TNF-α was also inhibited by fascin siRNA, implying that TNF-α-induced MMP-9 overexpression is mediated by fascin. Interestingly, they found that macrophages positive for TNF-α were commonly observed at the invasive front of cholangiocarcinoma compared with the central part of CC. Such locally released TNF- α from macrophages around the invasive front of CC may be responsible for such overproduction of fascin and then MMP-9. These findings may explain the correlated dense expression pattern of fascin and MMP-9 in breast carcinoma tissues at the invasive fronts, as shown in the current study.

Many previous studies have been conducted to research MMP-9 expression in human cancers, including breast cancer, but the results are still controversial. In the present study, MMP-9 expression was detected in 50.75% of breast carcinoma cases with weak expression in stromal cells. In contrast, the adjacent normal breast tissue did not express MMP-9. These results are consistent with Scorilas et al. [24] who observed MMP-9 staining primarily in cancer cells, and to a lesser degree in surrounding stromal cells but not in normal breast tissue. However, Pellikainen et al. [40] observed MMP-9 in both tumor cells and stromal fibroblasts and inflammatory cells. In addition, benign breast epithelium and vascular endothelium stained positively for MMP-9.

In the current research, 37.5% of invasive ductal carcinomas with adjacent in situ component revealed positive MMP-9 expression in the in situ carcinoma lesions. This result is in agreement with Kim et al. [41] who found MMP-9 mRNA expression in 50% of DCIS and 44% of invasive ductal carcinoma cases. They added that MMP mRNA expression levels suggest that an invasive potential of breast carcinoma is already obtained before morphologically overt invasive growth is observed.

In our study, there was a significant correlation between MMP-9 expression and lymph node metastases, advanced tumor stage as well as estrogen receptor negative and progesterone receptor negative hormonal status. Similar to these findings, Fan et al. [25] observed that MMP-9 overexpression was higher in breast cancers with lymph node metastases than those without lymph node metastases. They added that increased expression of MMP-9 protein was correlated with high TNM classification. Furthermore, Przybylowska et al. [42] and Slattery et al. [43] found a significant relationship between MMP-9 and ER-/PR- tumors. In addition, Liu et al. [44] observed a significant association between basal like breast cancer and MMP-9. In contrast, Zhang et al. [23] noted that high MMP-9 expression in tumor cells was not associated with any clinicopathological parameters or immunohistochemical expression of ER and PR. Scorilas et al. [24] found that MMP-9 negative tumors were obtained from patients who were diagnosed with stage III-IV disease and Grieu et al. [45] observed that MMP-9 21562 polymorphism was associated with ER positive tumors.

In the current research, no statistical association was detected between MMP-9 expression and patients’ age, tumor size, histological grade or HER2 status. Parallel to these results, Scorilas et al. [24] observed no significant association between MMP-9 and tumor grade and Zhang et al. [23] found no correlation between MMP-9 and c-erbB2 (HER2). In contrast, Li et al. [46] detected a significant relationship between positive MMP-9 immunostaining and higher tumor grade. Moreover, Fan et al. [25] and Przybylowska et al. [42] found that MMP-9 protein was positively associated with tumor size. The different results between our present study and others might be due to differences in sample size, methods of scoring criteria, and the antibodies used to evaluate expression.

Regarding breast cancer molecular subtypes, in the current research, triple negative breast cancers had the highest rate of fascin and MMP-9 expression. However, luminal breast cancer had the lowest rate of expression. It is well established in the literature that triple negative subtype is an independent prognostic factor of distant metastasis due to its strong invasive ability and metastasis ability [47]. It represents one of the most aggressive phenotype with discrete risk factors and ominous prognostic significance [48]. On the other hand, luminal subtype of breast cancer has better prognosis than other molecular subtypes [49]. Fascin and MMP-9 might be markers of aggressive behaviour in breast cancer.

## Conclusion

In conclusion, both fascin and MMP-9 proteins are associated with parameters of poor prognosis. Given fascin’s role in enhancing cell motility, the data presented here suggests that fascin expression may contribute to a more aggressive clinical course and thus an enhanced metastatic potential in ER/PR-negative breast cancer. Moreover, overexpression of MMP-9 might play a critical role in degradation of extracellular matrix to enhance the invasive and metastatic capacity of breast cancer. In addition, dense expression pattern of fascin and MMP-9 in breast carcinoma tissues at the invasive fronts confirms their role as pathogenic factors for tumor cell invasion. The current study reports for the first time the direct relationship between fascin and MMP-9 expression in breast cancer. This finding supports the role of fascin in cell invasiveness by activating matrix proteases besides increasing cell motility as postulated by previous studies. Finally, Fascin and MMP-9 may represent potential therapeutic targets for patients with breast cancer especially those with hormone receptor–negative status.

A limitation of this study is that the cases were derived from a regional population base that lacks breast cancer outcomes including response to different modalities of therapy and disease free survival. Therefore, further larger studies are still needed to explore the direct relation of facsin and MMP-9 expression to breast cancer patients’ survival and their prognostic value within different treatment modality subsets.

## Abbreviations

MMP-9: Matrix metalloproteinase-9; HCC: Hepatocellular carcinoma; MMP-2: Matrix metalloproteinase-2; ER: Estrogen receptor; PR: Progesterone receptor; CC: Cholangiocarcinoma; TNF-α: Tumour necrosis factor alpha; IHC: Immunohistochemistry; DCIS: Ductal carcinoma in situ.

## Competing interests

The authors declare that they have no competing interests.

## Authors’ contributions

NSY conceived, designed and coordinated the study, performed data collection, reviewed the histological diagnosis, evaluated immunohistochemistry, carried out photographing and drafted the manuscript. SAH reviewed the histological diagnosis, evaluated immunohistochemistry, participated in the study design, performed the statistical analysis, helped in photographing and critically reviewed the manuscript. The two authors read and approved the final manuscript.

## References

[B1] Chambers AF Naumov GN Varghese HJ Nadkarni KV MacDonald IC Groom AC Critical steps in hematogenous metastasis: an overview Surg Oncol Clin N Am 2001 10 243 255 11382585

[B2] Hashimoto Y Ito T Inoue H Okumura T Tanaka E Tsunoda S Higashiyama M Watanabe G Imamura M Shimada Y Prognostic significance of fascin overexpression in human esophageal squamous cell carcinoma Clin Cancer Res 2005 11 7 2597 2605 15814639 10.1158/1078-0432.CCR-04-1378

[B3] Hashimoto Y Skacel M Adams JC Roles of fascin in human carcinoma motility and signaling: prospects for a novel biomarker? Int J Biochem Cell Biol 2005 37 1787 1804 16002322 10.1016/j.biocel.2005.05.004

[B4] Kureishy N Sapountzi V Prag S Anilkumar N Adams JC Fascins and their roles in cell structure and function Bioessays 2002 24 350 361 11948621 10.1002/bies.10070

[B5] Adams JC Cell–matrix contact structures Cell Mol Life Sci 2001 58 371 392 11315186 10.1007/PL00000864 PMC11337345

[B6] Pinkus GS Pinkus JL Langhoff E Matsumura F Yamashiro S Mosialos G Said JW Fascin, a sensitive new marker for Reed– Sternberg cells of Hodgkin’s disease. Evidence for a dendritic or B cell derivation? Am J Pathol 1997 150 543 562 9033270 PMC1858289

[B7] Ozerhan IH Ersoz N Onguru O Ozturk M Kurt B Cetiner S Fascin expression in colorectal carcinomas Clinics (Sao Paulo) 2010 65 2 157 164 20186299 10.1590/S1807-59322010000200007 PMC2827702

[B8] Tong GX Yee H Chiriboga L Hernandez O Waisman J Fascin-1expression in papillary and invasive urothelial carcinomas of the urinary bladder Hum Pathol 2005 36 741 746 16084942 10.1016/j.humpath.2005.05.005

[B9] Maitra A Iacobuzio-Donahue C Rahman A Sohn TA Argani P Meyer R Yeo CJ Cameron JL Goggins M Kern SE Ashfaq R Hruban RH Wilentz RE Immunohistochemical validation of a novel epithelial and a novel stromal marker of pancreatic ductal adenocarcinoma identified by global expression microarrays: sea urchin fascin homolog and heat shock protein 47 Am J Clin Pathol 2002 118 52 59 12109856 10.1309/3PAM-P5WL-2LV0-R4EG

[B10] Pelosi G Pastorino U Pasini F Maissoneuve P Fraggetta F Iannucci A Sonzogni A De Manzoni G Terzi A Durante E Bresaola E Pezzella F Viale G Independent prognostic value of fascin immunoreactivity in stage I non small cell lung cancer Br J Cancer 2003 88 537 547 12592367 10.1038/sj.bjc.6600731 PMC2377175

[B11] Kim SJ Kim DC Kim MC Jung GJ Kim KH Jang JS Kwon HC Kim YM Jeong JS Fascin expression is related to poor survival in gastric cancer Pathol Int 2012 62 777 784 23252866 10.1111/pin.12012

[B12] Ortiz CM Ito T Hashimoto Y Nagayama S Iwai A Tsunoda S Sato F Martorell M Garcia JA Perez A Shimada Y Effects of small interfering RNAs targeting fascin on human esophageal squamous cell carcinoma cell lines Diagn Pathol 2010 5 41 20565981 10.1186/1746-1596-5-41 PMC2907320

[B13] Tan VY Lewis SJ Adams JC Martin RM Association of fascin-1 with mortality, disease progression and metastasis in carcinomas: a systematic review and meta-analysis BMC Med 2013 11 52 23442983 10.1186/1741-7015-11-52 PMC3635876

[B14] Grothey A Hashizume R Sahin AA McCrea PD Fascin, an actin-bundling protein associated with cell motility, is up regulated in hormone receptor negative breast cancer Br J Cancer 2000 83 870 873 10970687 10.1054/bjoc.2000.1395 PMC2374674

[B15] Yoder BJ Tso E Skacel M Pettay J Tarr S Budd T Tubbs RR Adams JC Hicks DG The expression of fascin, an actin-bundling motility protein, correlates with hormone receptor-negative breast cancer and a more aggressive clinical course Clin Cancer Res 2005 11 186 192 15671545

[B16] Rodriguez-Pinilla SM Sarrio D Honrado E Hardisson D Calero F Benitez J Palacios J Prognostic significance of basal-like phenotype and fascin expression in node-negative invasive breast carcinomas Clin Cancer Res 2006 12 1533 1539 16533778 10.1158/1078-0432.CCR-05-2281

[B17] Al-Alwan M Olabi S Ghebeh H Barhoush E Tulbah A Al-Tweigeri T Ajarim D Adra C Fascin is a key regulator of breast cancer invasion that acts via the modification of metastasis-associated molecules PLoS One 2011 6 e27339 22076152 10.1371/journal.pone.0027339 PMC3208623

[B18] Hayashi Y Osanai M Lee GH Fascin-1 expression correlates with repression of E-cadherin expression in hepatocellular carcinoma cells and augments their invasiveness in combination with matrix metalloproteinases Cancer Sci 2011 102 6 1228 1235 21323792 10.1111/j.1349-7006.2011.01910.x PMC11158138

[B19] Onodera M Zen Y Harada K Sato Y Ikeda H Itatsu K Sato H Ohta T Asaka M Nakanuma Y Fascin is involved in tumor necrosis factor-a-dependent production of MMP9 in cholangiocarcinoma Lab Invest 2009 89 1261 1274 19721413 10.1038/labinvest.2009.89

[B20] Xie JJ Xu LY Zhang HH Cai WJ Mai RQ Xie YM Yang ZM Niu YD Shen ZY Li EM Role of fascin in the proliferation and invasiveness of esophageal carcinoma cells Biochem Biophys Res Commun 2005 337 355 362 16185662 10.1016/j.bbrc.2005.09.055

[B21] Toi M Ishigaki S Tominaga T Metalloproteinases and tissue inhibitors of metalloproteinases Breast Cancer Res Treat 1998 52 113 124 10066076 10.1023/a:1006167202856

[B22] Kato Y Yamashita T Ishikawa M Relationship between expression of matrix metalloproteinase-2 and matrix metalloproteinase- 9 and invasion ability of cervical cancer cells Oncol Rep 2002 9 565 569 11956628

[B23] Zhang YG Du J Tian XX Zhong YF Fang WG Expression of E-cadherin, beta-catenin, cathepsin D, gelatinases and their inhibitors in invasive ductal breast carcinomas Chin Med J (Engl) 2007 120 18 1597 1605 17908479

[B24] Scorilas A Karameris A Arnogiannaki N Ardavanis A Bassilopoulos P Trangas T Talieri M Overexpression of matrix-metalloproteinase-9 in human breast cancer: a potential favourable indicator in node-negative patients Br J Cancer 2001 84 11 1488 1496 11384099 10.1054/bjoc.2001.1810 PMC2363667

[B25] Fan SQ Wei QY Li MR Zhang LQ Liang QC Expression and clinical significance of MMP-2, MMP-9, TIMP-1, and TIMP-2 in breast carcinoma Ai Zheng 2003 22 9 968 973 12969531

[B26] Thanakit V Sampatanukul P Ruangvejvorachai P Keelawat S The association of co-expression of CD44v4/MMP-9 with different nodal status in high-grade breast carcinoma patients J Med Assoc Thai 2005 88 Suppl 4 S30 S35 16622998

[B27] Singletary SE Allred C Ashley P Bassett LW Berry D Bland KI Borgen PI Clark GM Edge SB Hayes DF Hughes LL Hutter RV Morrow M Page DL Recht A Theriault RL Thor A Weaver DL Wieand HS Greene FL Staging system for breast cancer: revisions for the 6^th^ edition of the AJCC Cancer Staging Manual Surg Clin North A 2003 83 803 819 10.1016/S0039-6109(03)00034-3 12875597

[B28] Elston CW Ellis IO Pathological prognostic factors in breast cancer. I. The value of histological grade in breast cancer: experience from a large study with long-term follow-up Histopathology 1991 19 403 410 1757079 10.1111/j.1365-2559.1991.tb00229.x

[B29] Hsu SM Raine L Fanger H Use of avidin -biotin - peroxidase complex (ABC) in immunoperoxidase techniques: a comparison between ABC and unlabelled antibody (PAP) procedures J Histochem Cytochem 1981 29 577 580 6166661 10.1177/29.4.6166661

[B30] Cheng H Qin Y Fan H Su P Zhang X Zhang H Zhou G Overexpression of CARM1 in breast cancer is correlated with poorly characterized clinicopathologic parameters and molecular subtypes Diagn Pathol 2013 8 129 23915145 10.1186/1746-1596-8-129 PMC3766166

[B31] Insall RH Machesky LM Actin dynamics at the leading edge: from simple machinery to complex networks Dev Cell 2009 17 310 322 19758556 10.1016/j.devcel.2009.08.012

[B32] Woodhouse EC Chuaqui RF Liotta LA General mechanisms of metastasis Cancer 1997 80 1529 1537 9362419 10.1002/(sici)1097-0142(19971015)80:8+<1529::aid-cncr2>3.3.co;2-#

[B33] Jawhari AU Buda A Jenkins M Shehzad K Sarraf C Noda M Farthing MJ Pignatelli M Adams JC Fascin, an actin-bundling protein, modulates colonic epithelial cell invasiveness and differentiation in vitro Am J Pathol 2003 162 69 80 12507891 10.1016/S0002-9440(10)63799-6 PMC1851132

[B34] Esteva FJ Sahin AA Cristofanilli M Arun B Hortobagyi GN Molecular prognostic factors for breast cancer metastasis and survival Semin Radiat Oncol 2002 12 319 328 12382190 10.1053/srao.2002.35251

[B35] Alghanem AA Hussain S The effect of tumor size and axillary lymph node metastasis on estrogen and progesterone receptors in primary breast cancer J Surg Oncol 1986 31 218 221 3724175 10.1002/jso.2930310317

[B36] Clark GM McGuire WL Prognostic factors in primary breast cancer Breast Cancer Res Treat 1983 3 S69 S72 6689475 10.1007/BF01855130

[B37] Rochefort H Platet N Hayashido Y Derocq D Lucas A Cunat S Garcia M Estrogen receptor mediated inhibition of cancer cell invasion and motility: an overview J Steroid Biochem Mol Biol 1998 65 163 168 9699869 10.1016/s0960-0760(98)00010-7

[B38] Tong D Czerwenka K Sedlak J Schneeberger C Schiebel I Concin N Leodolter S Zeillinger R Association of in vitro invasiveness and gene expression of estrogen receptor, progesterone receptor, pS2 and plasminogen activator inhibitor-1 in human breast cancer cell lines Breast Cancer Res Treat 1999 56 91 97 10517346 10.1023/a:1006262501062

[B39] Grothey A Hashizume R Ji H Tubb BE Patrick CW Jr Yu D Mooney EE McCrea PD C-erbB-2/ HER-2 upregulates fascin, an actin-bundling protein associated with cell motility, in human breast cancer cell lines Oncogene 2000 19 4864 4875 11039904 10.1038/sj.onc.1203838

[B40] Pellikainen JM Ropponen KM Kataja VV Kellokoski JK Eskelinen MJ Kosma VM Expression of matrix metalloproteinase (MMP)-2 and MMP-9 in breast cancer with a special reference to activator protein-2, HER2, and prognosis Clin Cancer Res 2004 10 7621 7628 15569994 10.1158/1078-0432.CCR-04-1061

[B41] Kim HJ Park CI Park BW Lee HD Jung WH Expression of MT-1 MMP, MMP2, MMP9 and TIMP2 mRNAs in ductal carcinoma in situ and invasive ductal carcinoma of the breast Yonsei Med J 2006 47 3 333 342 16807982 10.3349/ymj.2006.47.3.333 PMC2688152

[B42] Przybylowska K Kluczna A Zadrozny M Krawczyk T Kulig A Rykala J Kolacinska A Morawiec Z Drzewoski J Blasiak J Polymorphisms of the promoter regions of matrix metalloproteinases genes MMP-1 and MMP-9 in breast cancer Breast Cancer Res Treat 2006 95 65 72 16267613 10.1007/s10549-005-9042-6

[B43] Slattery ML John E Torres-Mejia G Stern M Lundgreen A Hines L Giuliano A Baumgartner K Herrick J Wolff RK Matrix metalloproteinase genes are associated with breast cancer risk and survival: the Breast Cancer Health Disparities Study PLoS One 2013 8 e63165 23696797 10.1371/journal.pone.0063165 PMC3655963

[B44] Liu Y Xin T Jiang QY Huang DY Shen WX Li L Lv YJ Jin YH Song XW Teng C CD147, MMP9 expression and clinical significance of basal-like breast cancer Med Oncol 2013 30 366 23292863 10.1007/s12032-012-0366-x

[B45] Grieu F Li WQ Iacopetta B Genetic polymorphisms in the MMP-2 and MMP-9 genes and breast cancer phenotype Breast Cancer Res Treat 2004 88 197 204 15609121 10.1007/s10549-004-0595-6

[B46] Li HC Cao DC Liu Y Hou YF Wu J Lu JS Di GH Liu G Li FM Ou ZL Jie C Shen ZZ Shao ZM Prognostic value of matrix metalloproteinases (MMP-2 and MMP-9) in patients with lymph node-negative breast carcinoma Breast Cancer Res Treat 2004 88 75 85 15538048 10.1007/s10549-004-1200-8

[B47] Zhang P Xu BH Ma F Li Q Yuan P Wang JY Zhang P Treatment outcomes and clinicopathologic characteristics of advanced triple-negative breast cancer patients Chin J Oncol 2011 33 381 384 21875471

[B48] Hashmi AA Edhi MM Naqvi H Faridi N Khurshid A Khan M Clinicopathologic features of triple negative breast cancers: an experience from Pakistan Diagn Pathol 2014 9 43 24581278 10.1186/1746-1596-9-43 PMC3996046

[B49] Su Y Zheng Y Zheng W Gu K Chen Z Li G Cai Q Lu W Shu XO Distinct distribution and prognostic significance of molecular subtypes of breast cancer in Chinese women: a population-based cohort study BMC Cancer 2011 11 292 21749714 10.1186/1471-2407-11-292 PMC3157458

